# Cough Sensitivity to Several External Triggers is Associated with Multiple Non-respiratory Symptoms

**DOI:** 10.1007/s00408-023-00622-w

**Published:** 2023-05-08

**Authors:** Heikki O. Koskela, Johanna T. Kaulamo, Anne M. Lätti

**Affiliations:** 1 School of Medicine, University of Eastern Finland, Kuopio, Finland; 2 Unit for Medicine and Clinical Research, Pulmonary Division, Kuopio University Hospital, PL 100, 70029 KYS Kuopio, Finland

**Keywords:** Cough, Cough reflex, Cough hypersensitivity, Chronic cough, Somatoform disorders, Central nervous system sensitization

## Abstract

**Purpose:**

Enhanced responsiveness to external triggers is thought to reflect hypersensitivity of the cough reflex. It may involve an enhanced sensitivity of the afferent nerves in the airways and/or an abnormal processing of the afferent information by the central nervous system (CNS). The CNS processing of cough has been shown to involve the same regions as those in symptom amplification, a phenomenon that often manifests as multiple symptoms. The main purpose of the present study was to define whether the presence of several cough triggers is associated with multiple symptoms.

**Methods:**

2131 subjects with current cough responding to two email surveys filled in a comprehensive questionnaire about social background, lifestyle, general health, doctors’ diagnoses and visits, symptoms, and medication. Multiple symptoms was defined as three or more non-respiratory, non-mental symptoms.

**Results:**

A carefully controlled multiple regression analysis revealed that the number of cough triggers was the only cough characteristic associating with multiple non-respiratory, non-mental symptoms [aOR 1.15 (1.12–1.19) per one trigger, *p* < 0.001]. Among the 268 subjects with current cough both in the first survey and in the follow-up survey 12 months later, the repeatability of the trigger sum was good with an intraclass correlation coefficient of 0.80 (0.75–0.84).

**Conclusion:**

The association between the number of the cough triggers and multiple symptoms suggests that the CNS component of cough hypersensitivity may be a manifestation of non-specific alteration in the CNS interpretation of various body sensations. The number of cough triggers is a repeatable measure of cough sensitivity.

**Supplementary Information:**

The online version contains supplementary material available at 10.1007/s00408-023-00622-w.

## Introduction

Coughing in response to low levels of thermal, mechanical, or chemical triggers is nowadays regarded as the key clinical element in chronic cough. It is in the center of the concept ‘cough hypersensitive syndrome’ which has been suggested as an overarching diagnosis irrespective of the possible cough background disorders [1]. Furthermore, two recent data-driven analyses have revealed two cough phenotypes, the phenotype TBQ (several Triggers of cough, many cough Background disorders, and poor cough-related Quality of life), and the common phenotype. The presence of several triggers of cough was the most important cough characteristic to separate the two cough phenotypes. This phenotype can be recognized also among subjects with acute and subacute cough, in addition to chronic cough [2, 3].

Enhanced responsiveness to external triggers is thought to reflect hypersensitivity of the cough reflex arc [4, 5]. It may involve an enhanced sensitivity of the afferent nerves in the airways and/or abnormal processing of the afferent information by the central nervous system (CNS) [6]. The involvement of CNS is supported by functional brain imaging studies utilizing airway irritation by capsaicin, a potent cough provocation agent, which have found activity in right inferior frontal gyrus, insula, prefrontal cortex, and anterior and mid-cingulate cortex of the CNS [7–9]. Of note, patients with chronic cough manifest alterations in the function of these brain regions when compared to healthy subjects [7].

In our previous community-based studies, subjects with cough often reported multiple non-respiratory symptoms [10, 11]. This feature led to biologically unplausible, odd associations. For example, chronic cough was statistically significantly associated with toothache, headache, constipation etc. Presence of multiple symptoms is a key characteristic of a phenomenon called symptom amplification. It is a process whereby patients’ thoughts, emotions, and concerns heighten uncomfortable bodily sensations and symptoms, making them more salient, intense, unpleasant, and distressing. The mechanism behind this phenomenon is probably an altered processing of interoceptive stimuli in brain regions that impart cognitive and affective dimensions to physical sensations [12]. Of particular interest, these regions strongly overlap with those participating in the processing of cough-evoking stimulation by capsaicin. These findings suggest a mechanistic link between symptom amplification and cough hypersensitivity.

The main purpose of the present study was to define whether the presence of several cough triggers is associated with multiple non-respiratory symptoms. The second purpose was to investigate the long-term repeatability of the cough triggers.

## Materials and Methods

### Population

**Fig. 1:**
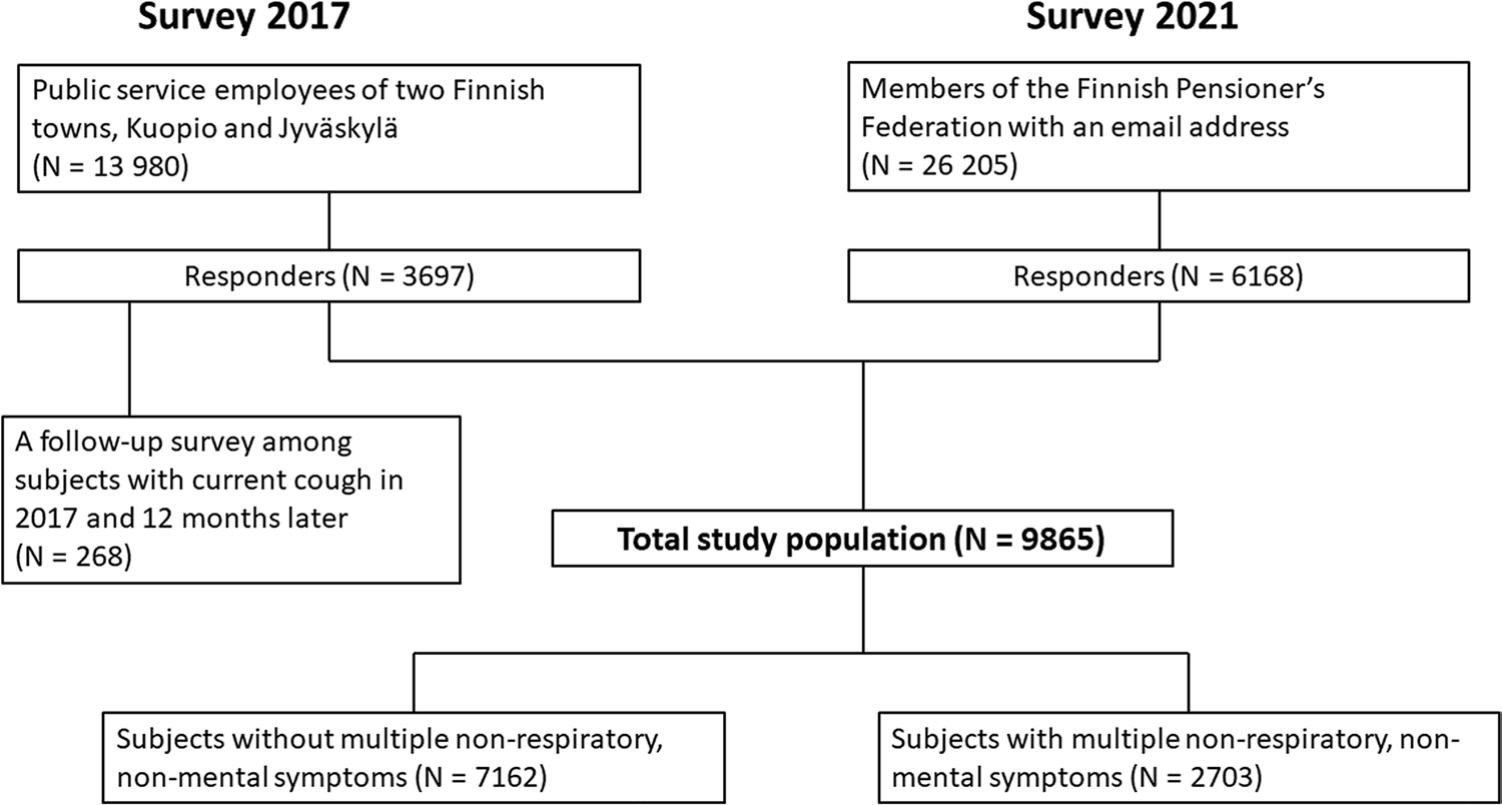
The flow chart

The population of the present study consists of the responders to two large population-based e-mail surveys originally planned to investigate the risk factors, prevalence, characteristics, and consequences of cough (Fig. 1) [10, 11]. The first survey was performed in spring 2017. The target population was the public service employees of two middle-sized towns in central Finland, Jyväskylä and Kuopio. The response rate was 26% [10]. The second one was performed in spring 2021. The target population was the members of the Finnish Pensioner’s Federation. The response rate was 24% [11]. In both studies, the sex and age distribution of the respondents closely resembled those of the target populations. Altogether, there were 9865 well-characterized subjects with an age range of 18–94 years (Table 1).

**Table 1:** The characteristics of the 9865 subjects, divided whether they have or have not multiple non-respiratory, non-mental symptoms (MNNS)

Characteristic	Without MNNS (*N* = 7162)	With MNNS (*N* = 2703)	*p* value
Age (years)	63.9 (63.6–64.3)	61.0 (60.4–61.5)	< 0.001
Body mass index (kg/m^2^)	26.8 (26.6–26.9)	28.1 (27.9–28.3)	< 0.001
Female gender (%)	69.5	80.6	< 0.001
Length of education (years)	14.0 (13.9–14.1)	14.2 (14.0–14.3)	0.001
Yearly gross family income^a^ (euros)	40,000–70,000	15,000–40,000	0.003
Living alone (%)	18.9	20.4	0.085
Yearly doctor’s visits due to any reason	2.24 (2.18–2.31)	3.70 (3.51–3.89)	< 0.001
Number of disorders diagnosed by a doctor	1.06 (1.03–1.09)	1.71 (1.65–1.77)	< 0.001
Depressive symptoms (%)	4.0	7.3	< 0.001
Current cough (%)	17.8	31.7	< 0.001

The figures are means and 95% confidence intervals or percentiles unless stated otherwise

^a^Median value of the following five alternatives: below 15,000, 15,000–40,000, 40,000–70,000, 70,000–120,000, and over 120,000

The subjects with current cough (cough within the past 2 weeks) in the spring 2017 survey were sent a shorter follow-up questionnaire 12 months later. The response rate was now 75.3%. The triggers were asked, again, and the repeatability of the trigger sum and individual triggers were thereby analyzed. Of note, the cough was not necessarily continuous throughout the 12 months’ period but was interrupted in many subjects.

The decision to respond was considered as an informed consent. The study was approved by the Ethics Committee of Kuopio University Hospital (289/2015). Permissions to conduct the surveys were obtained from the officials of Jyväskylä and Kuopio and from the Finnish Pensioners’ Federation.

### The Questionnaires

The questionnaires of the surveys in 2017 and 2021 were almost identical but slightly modified due to the differences in the two populations’ age and employment status [10, 11]. Both questionnaires included questions about social background, lifestyle, general health, doctors’ diagnoses and visits, symptoms and medication. Validated questions for depressive symptoms were included [13]. Respondents with current cough answered additional cough-related questions including the Leicester Cough Questionnaire [14]. All surveys included a list of ten external triggers (Table 2) and the year 2021 survey also four allotussia-type triggers (supplementary Information, Table 1).

**Table 2:** The ten external triggers asked in all surveys from the subjects with current cough

Trigger	Proportion of subjects reporting the trigger (*N* = 2131)	Adjusted OR (95% CI) for MNNS (*N* = 2131)	*p* value for the association with MNNS (*N* = 2131)	Kappa value as a measurement of agreement 12 months apart (*N* = 268)
Upper respiratory tract infection	52.3%	1.74 (1.44–2.10)	< 0.001	0.27
Subfreezing air	36.0%	1.87 (1.54–2.26)	< 0.001	0.56
Physical exercise	23.5%	1.68 (1.36–2.08)	< 0.001	0.50
Automobile exhaust fumes	28.0%	1.80 (1.47–2.21)	< 0.001	0.51
Poor indoor air quality	38.9%	1.88 (1.56–2.28)	< 0.001	0.57
Proximity to animals	12.0%	1.67 (1.27–2.20)	< 0.001	0.53
Pollens	30.0%	1.50 (1.23–1.83)	< 0.001	0.60
Cigarette smoke	30.2%	1.66 (1.37–2.03)	< 0.001	0.58
Strong scents	37.2%	1.75 (1.45–2.12)	< 0.001	0.68
Strong paints or fumes	34.6%	1.72 (1.42–2.09)	< 0.001	0.57

*MNNS* multiple non-respiratory symptoms

### Definitions

Multiple non-respiratory, non-mental symptoms (MNNS) was defined as presence of at least three symptoms from a list consisting of 10 common non-respiratory, non-mental symptoms: chest pain on exertion, aching joints, back pain, sciatica (back pain that radiates to the leg), toothache, swollen feet, headache, constipation, other gut problems (flatulence, diarrhea), and urinary problems. The cut-off value three was chosen to indicate the highest tertile in the total population of 9865 subjects. Disorder sum was calculated by summing up the reported disorders diagnosed by a doctor from a list of 15 non-respiratory, non-mental disorders (supplementary Information, Table 2). Trigger sum was calculated by summing up the reported cough triggers from a list of the ten external triggers, which were asked in all surveys (Table 2). Sputum production was assessed utilizing the LCQ question about it (In the last 2 weeks, have you been bothered by sputum (phlegm) production when you cough? There were 7 alternative answers from ‘every time’ to ‘never’). Presence of depressive symptoms was defined as a Patient Health Questionnaire-2 score of three or more [13].

### Statistical Analysis

Mann–Whitney *U* test or chi-square test were used to compare the groups. The multivariate analyses were conducted using binary logistic regression with a backward directed stepwise process. The dependent variable was the presence of MNNS. The independent variables were chosen on the basis of statistically significant association with MNNS in the bivariate analysis. The repeatability of the cough triggers and the trigger sum were analyzed utilizing the intraclass correlation coefficient, Bland–Altman plot and the Kappa value [15, 16]. SPSS software version 26.0 (IBM SPSS Statistics for Windows) was utilized and a *p*-value < 0.05 was considered statistically significant. The data is expressed as means and 95% confidence intervals (CI) unless stated otherwise.

## Results

In the total population, the 7162 subjects without MNNS and the 2703 subjects with MNNS differed in many respects (Table 1). Also, among the 2131 subjects with current cough, those with and without MNNS differed significantly in terms of many background characteristics in the bivariate analyses (Table 3). Of the various cough characteristics, sputum production, presence of any cough trigger, trigger sum, and the LCQ scores differed significantly between the groups. On the contrary, cough bout frequency and the length of the current cough episode did not associate with MNNS. The subjects with MNNS visited a doctor due to cough more often than those without.

**Table 3:** The characteristics of the 2131 subjects with current cough, divided whether they have or have not multiple non-respiratory, non-mental symptoms (MNNS)

Characteristic	Without MNNS (*N* = 1274)	With MNNS (*N* = 857)	*p* value
Age (years)	62.0 (61.2–62.7)	60.9 (59.9–61.9)	0.08
Body mass index (kg/m^2^)	27.1 (26.9–27.4)	28.3 (28.0–28.7)	< 0.001
Female gender (%)	70.8	81.4	< 0.001
Length of education (years)	14.3 (14.0–14.6)	14.3 (14.0–14.5)	0.44
Yearly gross family income^a^ (euros)	40,000–70,000	40,000–70,000	0.14
Living alone (%)	18.5	19.7	0.49
Number of disorders diagnosed by a doctor	1.19 (1.12–1.26)	1.79 (1.69–1.89)	< 0.001
Depressive symptoms (%)	5.30	9.30	< 0.001
Yearly doctor’s visits due to cough	0.69 (0.61–0.77)	0.89 (0.78–1.01)	0.001
Cough bout frequency^b^	Every day at least once a day	Every day at least once a day	0.45
Length of the current cough episode^c^	More than 2 months, but less than 1 year	More than 2 months, but less than 1 year	0.12
Sputum production when coughing^d^	Some times	Some times	0.004
Presence of any cough trigger	73.1	83.1	< 0.001
Trigger sum	2.69 (2.54–2.84)	4.03 (3.82–4.23)	< 0.001
LCQ physical domain	5.15 (5.10–5.20)	4.80 (4.74–4.87)	< 0.001
LCQ psychological domain	4.99 (4.93–5.05)	4.80 (4.72–4.88)	0.001
LCQ social domain	5.40 (5.34–5.46)	5.16 (5.08–5.24)	< 0.001
LCQ total score	15.5 (15.4–15.7)	14.8 (14.5–15.0)	< 0.001

The figures are means and 95% confidence intervals or percentiles unless stated otherwise

*LCQ* Leicester Cough Questionnaire

^a^Median value of the following alternatives: 1. below 15,000, 2. 15,000–40,000, 3. 40,000–70,000, 4. 70,000–120,000, and 5. over 120,000

^b^Median value of the following alternatives: 1. several times a day, 2. every day at least once a day, 3. 4–6 days in a week, 4. two or three times a week, 5. at least once every week, 6. less than weekly

^c^Median value of the following alternatives: 1. less than 1 week, 2. longer than 1 week, but less than 3 weeks, 3. more than 3 weeks, but less than 2 months, 4. more than 2 months, but less than 1 year, 5. more than 1 year, but less than 5 years, 6. more than 5 years, but less than 10 years, 7. more than 10 years

^d^Median value of the following alternatives: 1. every time, 2. most times, 3. several times, 4. some times, 5. occasionally, 6. rarely, 7. never. Though the median values were the same, those with MNNS reported more often sputum production (Mann–Whitney test)

**Fig. 2:**
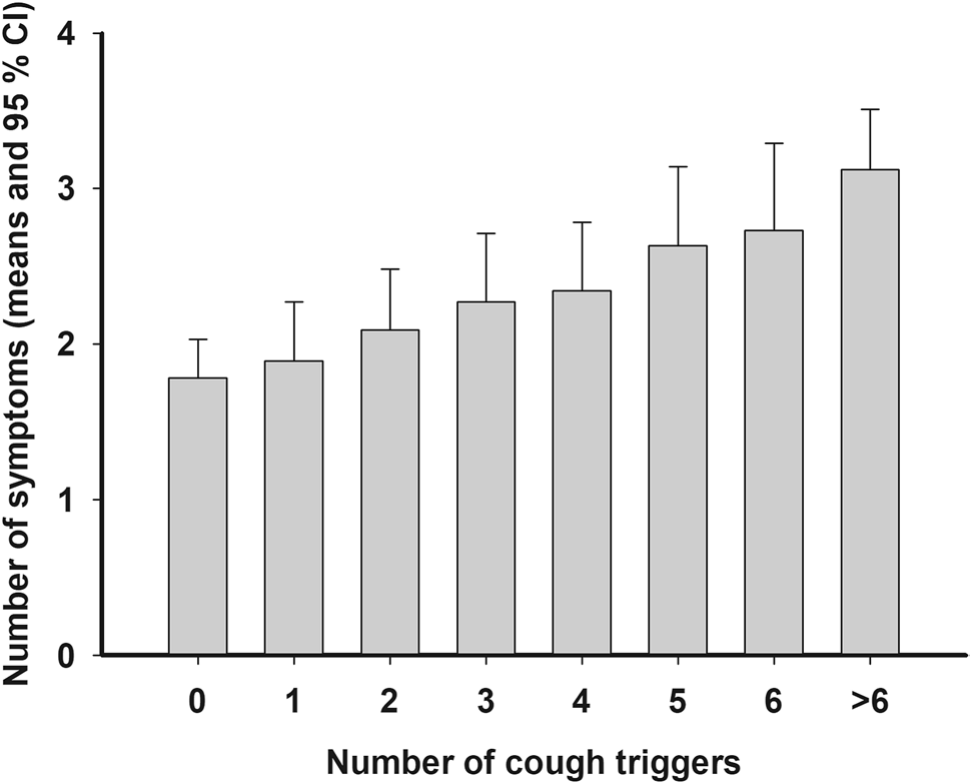
The relation between the number of cough triggers and the number of non-respiratory, non-mental symptoms among 2131 subjects with current cough

The multivariate analysis among the subjects with current cough included the following independent variables: body mass index, gender, presence of depressive symptoms, disorder sum, sputum production, trigger sum, and LCQ total score. Trigger sum was the only cough characteristic that showed an independent, statistically significant association with MNNS (Table 4, Fig. 2). When trigger sum was replaced by the presence of any trigger in this model, its adjusted OR was 1.58 (1.26–1.99, *p* < 0.001). All ten external triggers were also statistically significantly associated with MNNS (Table 2). The four allotussia-type triggers, which were used only in the year 2021 survey, were less strongly associated with MNNS (Supplementary Information, Table 1).

**Table 4:** Association of the various characteristics with multiple non-respiratory, non-mental symptoms (MNNS) among the 2131 subjects with current cough according to multiple logistic regression analysis

Characteristic	Adjusted odds ratio with 95% confidence intervals	*p* value
Trigger sum	1.15 (1.12–1.19) per one trigger	< 0.001
Female gender	1.80 (1.43–2.27)	< 0.001
Disorder sum	1.36 (1.27–1.46) per one disorder	< 0.001
Body mass index	1.03 (1.01–1.05) per one kg/m^2^	0.004
Depressive symptoms	1.53 (1.06–2.21)	0.023

**Fig. 3:**
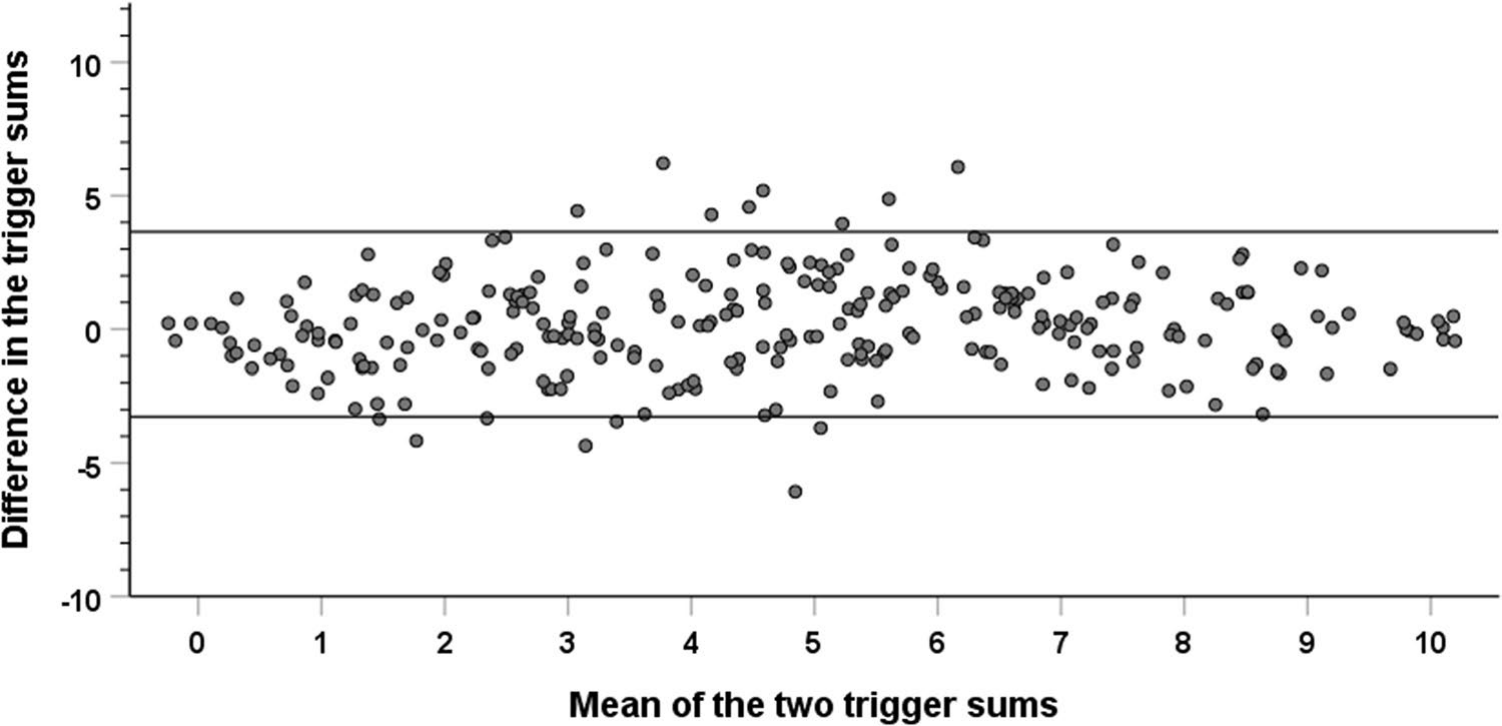
A Bland–Altman plot to demonstrate the repeatability of the trigger sum in 12 months’ interval among 268 subjects with current cough at both time-points. The difference of the first trigger sum minus the second trigger sum is plotted against the mean of the two trigger sums. The two horizontal lines indicate mean ± 1.96 SD of the difference between the trigger sums

Among the 268 subjects with current cough both in the spring 2017 survey and in the survey 12 months later, the repeatability of the trigger sum was good with an intraclass correlation coefficient of 0.80 (0.75–0.84). The repeatability was not affected by the number of reported triggers (Fig. 3). The Kappa value for any trigger was 0.30. The kappa values for all triggers are expressed in Table 2.

## Discussion

In this large, community-based population with a wide age range, the number of cough triggers showed a statistically highly significant, independent association with MNNS. This finding supports the idea that cough responsiveness to several triggers may share similar pathophysiological mechanisms with symptom amplification. Furthermore, the present study for the first time reports the good long-term repeatability of the trigger sum.

As mentioned, airway irritation by capsaicin evokes activity in right inferior frontal gyrus, insula, prefrontal cortex, and anterior and mid-cingulate cortex of the CNS and patients with chronic cough manifest alterations in the function of these brain regions when compared to healthy subjects [7–9]. These CNS regions are also activated by a wide range of other body sensations like desire to void in response to bladder dilatation [17], pain, discomfort and urge-to-defecate by rectal distension [18], pain due to esophageal distension [19], breathlessness sensation due to resistive inspiratory loading [20], and palpitation sensation induced by isoproterenol [21]. In addition, these regions show abnormal activation among subjects with medically unexplained symptoms, i.e., somatoform disorders [12]. They impart cognitive and affective dimensions to body sensations and an altered function of them is thought to be the key pathophysiological basis for symptom amplification [12]. Clinically, this may manifest, for example, as a disproportionate symptom stress, non-specific medication side effects, and symptoms that persist despite adequate medical therapy. One of the cardinal features of symptom amplification is multiplicity of symptoms [12, 22]. We propose that the presented strong association between multiple cough triggers and MNNS indicate overlapping interpretative CNS mechanisms in cough reflex hypersensitivity and symptom amplification.

Of note, the list of symptoms to define MNNS did not include respiratory or mental symptoms, to exclude any plausible biological mechanisms that could link the symptoms with cough. For example, wheezing is typical for asthma, which also causes prolonged cough [10]. Depressive symptoms, in turn, can develop in long-standing cough as a secondary phenomenon [23]. Thus, it is unlikely that the symptoms that defined MNNS can relate to multiple cough triggers by other mechanisms than an abnormal CNS interpretation of body sensations, i.e., symptom amplification.

To the best of our knowledge, there are no previous studies investigating the repeatability of the reported cough triggers. In the present study, the long-term repeatability of trigger sum was good when measured twice 12 months apart. Furthermore, the repeatability was unaffected by the number of triggers. On the contrary, the repeatability of individual triggers was unsatisfactory, suggesting that the sum of triggers is the preferable way to clinically evaluate cough sensitivity. It seems that the subjective importance of individual cough triggers may vary with time but the tendency to experience them, as expressed in the trigger sum, is more constant.

An enhanced responsiveness to cough triggers is widely considered a clinical correlate for cough hypersensitivity [4, 5] and in the present study, the number of subject-reported cough triggers was assumed to reflect the degree of cough reflex arc hypersensitivity. However, the objective evidence about this association is scarce. Strong scents [24–29] and cold air [30] as cough triggers are associated with an enhanced cough response to capsaicin. In addition, objectively documented coughing in response to deep inspiration is associated with cough hypersensitivity to citric acid [31]. However, we are unaware about studies correlating the number of cough triggers with the degree of objectively measured cough sensitivity. Thus, the number of cough triggers needs more validating to be used as a clinical correlate for cough reflex arc hypersensitivity.

Another shortcoming in the present study is that the allotussia-type triggers were only asked in the year 2021 survey and were therefore not included in the trigger sum. However, these triggers were found to be less associated with MNNS than the external triggers and therefore, this shortcoming may not invalid the findings. Low response rate may also be regarded as a weakness. It is possible that subjects with more troublesome cough are over-represented. However, the age and the gender distributions of the responders resembled closely those of the original populations, as reported before [10, 11]. Finally, our study was a survey and objective tests to measure cough reflex sensitivity were not included.

The strengths of the present study include the large, well characterized population. Most of the characteristics that have shown to associate with multiple symptoms could be considered in the multivariate models and therefore, the independent association between the trigger sum and MNNS could be demonstrated with a great certainty. In particular, many other studies have not controlled adequately for general medical disorders. They form an important predictor of multiple symptoms: the more general medical disorders a person has, the more symptoms he/she probably reports [32]. This was considered in the present study by utilizing the variable ‘Disorder sum’.

In conclusion, the strong association between the number of the reported cough triggers and MNNS suggests that the CNS component of cough hypersensitivity may not be a specific phenomenon for cough, but a manifestation of a wider, non-specific alteration in the CNS interpretation of various body sensations, i.e., symptom amplification. This hypothesis is supported by functional brain imaging studies that have revealed overlapping functional CNS regions involved in the phenomena [7–9, 12]. This finding may provide a novel treatable trait for chronic cough, since subjects with symptom amplification have benefited from chronic disease management programs, cognitive behavior therapy, and psychotherapy [12]. The association of the cough hypersensitivity with symptom amplification may also clarify the mechanisms of the extraordinary high placebo effect of cough medications and the relatively modest effect of the new P2X3 antagonists, which act on peripheral afferent nerves only [33].

## Supplementary Information

Below is the link to the electronic supplementary material.

Supplementary file1 (DOCX 23 KB)
