# Supplementary material for: Cough Sensitivity to Several External Triggers is Associated with Multiple Non-respiratory Symptoms
Source: Lung. 2023 May 8;201(3):267–74. doi: 10.1007/s00408-023-00622-w (PMC10284963; doi:10.1007/s00408-023-00622-w)
Supplement: Supplementary file 1 — Supplementary file1 (DOCX 23 KB) [file 408_2023_622_MOESM1_ESM.docx]

Supplementary Tables

**Cough sensitivity to several external triggers is associated with multiple non-respiratory symptoms**

The second, revised version

Submitted to: LUNG

Heikki O Koskela^1,2^,MD, PhD, Johanna T Kaulamo^1^, MD, Anne M Lätti^2^, MD, PhD

**Affiliation of the authors**

^1^School of Medicine, University of Eastern Finland, Kuopio, Finland.

^2^Unit for Medicine and Clinical Research, Pulmonary Division, Kuopio University Hospital, Kuopio, Finland.

**Corresponding author:** Heikki Koskela, Unit for Medicine and Clinical Research, Pulmonary Division, Kuopio University Hospital, Kuopio, Finland. Postal address: PL 100, 70029 KYS, FINLAND

Supplementary table 1. The four allotussia-type cough triggers, which were asked in the year 2021 survey from 1109 elderly subjects with current cough.

| **Trigger** | **Proportion of subjects reporting the trigger** | **Adjusted OR**  **(95 % CI) for MNNS** | **P value for the association with MNNS** |
| --- | --- | --- | --- |
| Speaking | 22.4 % | 1.17 (0.86 – 1.58) | 0.32 |
| Eating/drinking | 21.0 % | 1.51 (1.11 – 2.05) | 0.008 |
| Laughing | 12.3 % | 1.32 (0.90 – 1.94) | 0.15 |
| Deep inspiration | 12.7 % | 1.03 (0.71 – 1.51) | 0.88 |

MNNS = multiple non-respiratory, non-mental symptoms.

Supplementary table 2. The list of 15 non-respiratory, non-mental disorders diagnosed by a doctor, which were asked from the 2131 subjects with current cough, and utilized to calculate the disorder sum.

| Gastric distress (gastritis, gastric ulcer) |
| --- |
| Esophageal reflux disease (heartburn, gastroesophageal reflux) |
| Sleep apnea |
| Parkinson’s disease |
| Rheumatoid arthritis |
| Other connective tissue disorders (Sjögren’s syndrome etc.) |
| Hypothyreosis |
| Arterial hypertension |
| Elevated blood cholesterol |
| Diabetes |
| Myocardial infarction |
| Coronary artery disease, angina pectoris |
| Cancer |
| Spinal disc problems, other back disease |
| Prostate hyperplasia |
